# From bytes to nephrons: AI’s journey in diabetic kidney disease

**DOI:** 10.1007/s40620-024-02050-2

**Published:** 2024-08-12

**Authors:** Debargha Basuli, Akil Kavcar, Sasmit Roy

**Affiliations:** 1 Department of Nephrology & Hypertension, Brody School of Medicine, East Carolina University, 2355 W Arlington Blvd, Greenville, NC 27834 USA; 2 Department of Internal Medicine, Brody School of Medicine, East Carolina University, Greenville, NC USA; 3 Department of Nephrology, University of Virginia, Lynchburg, VA USA

**Keywords:** Artificial intelligence, Machine learning, Diabetic kidney disease, Chronic Kidney Disease

## Abstract

Diabetic kidney disease (DKD) is a significant complication of type 2 diabetes, posing a global health risk. Detecting and predicting diabetic kidney disease at an early stage is crucial for timely interventions and improved patient outcomes. Artificial intelligence (AI) has demonstrated promise in healthcare, and several tools have recently been developed that utilize Machine Learning with clinical data to detect and predict DKD. This review aims to explore the current landscape of AI and machine learning applications in DKD, specifically examining existing literature on risk scores and machine learning approaches for predicting DKD development. A literature search was conducted using Medline (PubMed), Google Scholar, and Scopus databases until July 2023. Relevant keywords were used to extract studies that described the role of AI in DKD. The review revealed that AI and machine learning have been successfully used to predict DKD progression, outperforming traditional risk score models. Artificial intelligence-driven research for DKD extends beyond prediction models, offering opportunities for integrating genetic and epigenetic data, advancing understanding of the disease’s molecular basis, personalizing treatment strategies, and fostering the development of novel drugs. However, challenges remain, including the requirement for large datasets and the lack of standardization in AI-driven tools for DKD. Artificial intelligence and machine learning have the potential to revolutionize the management and care of DKD patients, surpassing the limitations of traditional methods reliant on existing knowledge. Future research should address the challenges associated with AI and machine learning in DKD and focus on developing AI-driven tools for clinical practice.

**Graphical abstract:**

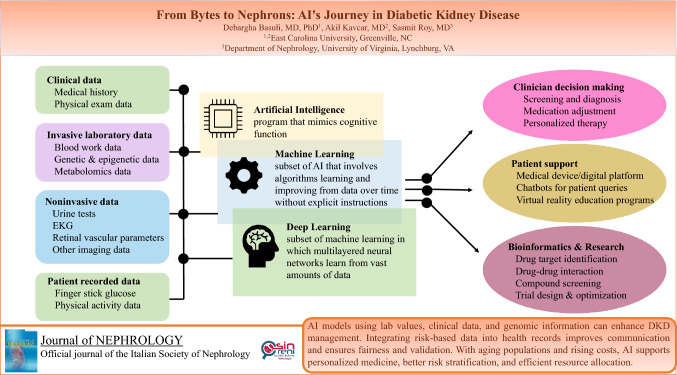

## Introduction

Diabetic kidney disease (DKD), also known as diabetic nephropathy, is a common and significant complication of type 2 diabetes (T2D). It accounts for a majority of the increased risk of mortality among patients with diabetes [1]. The global prevalence of DKD is increasing, and it is estimated that over 400 million people worldwide have the disease. Diabetic kidney disease is a progressive disease, and once it develops, it is considered to be irreversible. Early detection and prediction of DKD are crucial for implementing timely interventions and preventing disease progression. Routine screening for DKD, based on clinical and laboratory measurements, is not universally implemented, leading to missed or delayed diagnoses. Therefore, there is a need for accurate risk stratification and prediction models to identify high-risk patients, particularly those newly diagnosed with T2D.

Artificial intelligence (AI) and machine learning approaches have emerged as powerful tools in healthcare, offering the potential to improve diagnostics, prognostics, and treatment decisions. Machine learning algorithms can analyze large amounts of clinical data, genetic information, and imaging findings to develop predictive models for disease outcomes [2]. However, while machine learning has shown promise in various medical fields, its application in predicting DKD progression and complications in patients with T2D is still limited. There is a need for more precise predictive models that can enable early interventions in DKD and prevent further progression in patients without apparent symptoms or signs. Encouragingly, a surge of private enterprises has surfaced, actively developing real-world solutions that harness the power of machine learning methods to identify individuals at risk of chronic kidney disease (CKD), including DKD. Notably, the recent approval of KidneyIntelX by the U.S. Food and Drug Administration (FDA) serves as a resounding testament to its potential in the diagnosis and management of rapidly progressive kidney disease in individuals with type 2 diabetes, underscoring the transformative impact of AI in this domain.

This review article aims to explore the current landscape of AI and machine learning applications specifically in DKD. We will examine the existing literature, including risk scores and machine learning approaches, for predicting DKD development and progression. Additionally, we will discuss the challenges and opportunities in utilizing AI and machine learning in the context of DKD and highlight the potential for personalized medicine and improved patient outcomes. By critically assessing the available evidence and discussing the limitations and future directions, this review aims to provide insights into the role of AI and machine learning in transforming the management and care of patients with DKD.

## Methodology

**Fig. 1:**
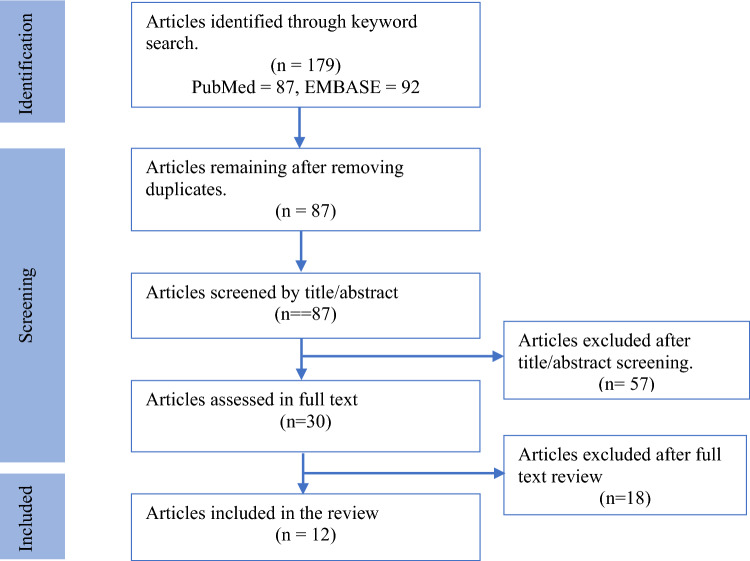
Study selection flow chart

We performed a comprehensive systematic review of articles focusing on the use of AI or machine learning on DKD within the last decade, limiting our search to English language publications. We conducted our search across two major electronic databases, PubMed MEDLINE and EMBASE, up to December 31, 2023. Employing a combination of relevant key terms and synonyms, including “diabetic kidney disease”, “artificial intelligence”, and “machine learning” and aliases (other MeSH and emtree terms), we meticulously screened retrieved articles. This process began with the removal of duplicates, followed by independent abstract assessments by three authors. Discrepancies in article selection were resolved through consensus. Subsequently, selected articles underwent full-text examination to make the final inclusion decisions. Reference lists of included studies were also reviewed for relevant articles. In total, our systematic search identified 12 studies meeting our criteria, where machine learning protocols were used in DKD (Fig. 1).

## Advancements in predictive modeling for diabetic kidney disease progression: recent studies and findings

In recent years, some studies have shown significant promise in predicting genotype–phenotype risk patterns and quantifying the risk of CKD as a complication of diabetes. Some of the more important studies, categorized based on the function of the AI, are summarized below (Table 1).

**Table 1:** Summary of Key Studies Utilizing Artificial Intelligence/Machine Learning in Diabetic Kidney Disease Research

Study	Methodology	Key Findings
Prediction		
Leung et al. 2012 [3]	ML and mathematical modeling	Age, age of diagnosis, and lipid parameters were important clinical indicators for DKD. Genetic polymorphisms linked to inflammation and lipid metabolism were influential genetic predictors
Allen et al. 2020 [4]	ML algorithm using EHR	ML models outperformed the CDC risk score in predicting the development of DKD in patients with T2D
Makino et al. 2019 [5]	AI and EMR data analysis	AI and EMR data were used to develop a predictive model for DKD progression. The model achieved a 71% accuracy in identifying time series patterns related to DKD aggravation
Dong et al. 2021 [6]	Comparison of various ML algorithms	The LightGBM model showed the best predictive performance for 3-year DKD risk in patients with T2D and normoalbuminuria. Important risk factors included age, homocysteine, hemoglobin A1c, and eGFR
Risk Quantification		
Ravizza et al. 2019 [7]	Comparison of algorithms trained on real world data and clinical data	Algorithms trained on real-world data achieved comparable or improved accuracy in quantifying the risk of DKD
Chan et al. 2021 [8]	ML risk score using biomarker data and EHR information	The developed KidneyIntelX risk score demonstrated superior performance in predicting DKD progression compared to models based solely on standard clinical variables. Significant improvements in discrimination and positive predictive value were observed, aligning with the goals of initiatives aiming to prevent or slow DKD progression
Early Detection		
Holmstrom et al. 2022 [11]	ECG-based deep learning models	ECG-based deep learning models accurately identified all stages of CKD, particularly in patients under 60 years of age. Favorable discrimination accuracy was observed in high-risk subgroups, including patients with diabetes and hypertension
Song et al. 2021 [14]	Ensemble feature selection framework	Previously unconsidered features such as serum anion gap and drug-drug interactions were found to be associated with DKD risk. The framework improved predictability and stability in identifying features associated with DKD
Shi et al. 2023 [12]	Comparison of various ML algorithms	A novel ML algorithm for DKD diagnosis based on fundus images indicated that retinal vascular changes could assist in DKD screening and detection
Biomarker Identification		
Hirakawa et al. 2022 [13]	Deep learning analysis of metabolomics data	Deep learning methods identified potential biomarkers for predicting DKD progression, including systolic blood pressure, urinary albumin-to-creatinine ratio, and several metabolites
R Wu et al. 2022 [15]	XGBoost and Random Forest	Serum myoglobin was identified as a novel biomarker of DKD, with elevated levels associated with increased DKD risk and mediation of renal impairment caused by metabolic syndrome components
Novel Drug Target Identification		
Abedi et al. [16]	Developed novel ML algorithm, mGMDH-AFS, for drug target prediction	Identified 13 differentially expressed microRNAs in the kidney cortex and 6 in the medulla as potential targets in DKD

*DKD* diabetic kidney disease, *ML* machine learning, *EHR* electronic health record, *AI* artificial intelligence, *EMR* electronic medical record, *LightGBM* light gradient boosting machine, *T2D* type 2 diabetes, *ECG* electrocardiogram, *CKD* chronic kidney disease, *XGBoost* extreme gradient boosting, *mGMDH-AFS* modified group method of data handling - adaptive feature selection

## Prediction

Predicting DKD in patients with T2D has evolved remarkably with the advent of machine learning techniques. By harnessing the vast potential of electronic health records (EHR) data, researchers have been able to identify key risk factors and improve early diagnosis and management of this severe complication. The journey of these advancements tells a compelling story of innovation and more in-depth understanding in the medical field.

In the early 2010s, one of the pioneering studies using machine learning to predict DKD utilized a multi-staged approach that combined machine learning with mathematical modeling [3]. This study highlighted crucial clinical indicators such as age, age at diagnosis, and lipid parameters, while also bringing genetic polymorphisms linked to inflammation and lipid metabolism to the forefront as significant genetic predictors of DKD. This work laid a robust foundation for future research, showcasing the potential of machine learning in identifying complex risk patterns. Building upon this foundation, another study pushed the boundaries further by leveraging a large dataset from over 700 healthcare sites across the US, spanning from 2007 to 2020 [4]. The study involved 1,200 patients with T2D who had been followed for at least 5 years. By employing advanced machine learning algorithms, specifically random forests (RF) and gradient-boosted trees (XGB), they developed a predictive model that achieved an area under curve (AUC) of 0.75 for predicting any stage of CKD and 0.82 for predicting more severe stages (3–5). The model was validated on a hold-out dataset of 200 patients. The authors concluded that their machine learning algorithm model outperformed the Centers for Disease Control and Prevention (CDC) risk score in both the hold-out test and external datasets. In a complementary study from Japan, the researchers introduced a novel approach by incorporating structured, text, and longitudinal data from electronic medical records (EMRs) [5]. Their approach utilized a convolutional autoencoder and a random forests classifier to capture time series patterns related to DKD aggravation over 6 months. Achieving an AUC of 0.743 and 71% accuracy, their model provided critical insights into the progression of DKD and its long-term implications, such as the increased likelihood of requiring hemodialysis within 10 years for patients with aggravated DKD. These last two studies underscored the importance of considering a broad spectrum of data types to enhance predictive models’ robustness. Adding another layer of complexity and innovation, Dong et al. [6] focused on T2D patients with normoalbuminuria, a subgroup not traditionally linked with increased DKD risk. By comparing various machine learning algorithms, they identified the light gradient boosting machine (LightGBM) model as the most effective in predicting 3-year DKD risk. Their findings brought to light new risk factors such as homocysteine, albumin, and bicarbonate levels, along with traditional markers like hemoglobin A1c and body mass index. This study not only improved predictive accuracy but also challenged existing paradigms by highlighting previously overlooked risk factors, contributing novel insights to the field.

The cumulative narrative of these studies illustrates a journey from early explorations to sophisticated, multifaceted models that significantly enhance our ability to predict and manage DKD in T2D patients. Each study built on the previous ones, incorporating more data, refining algorithms, and uncovering new risk factors, thereby pushing the boundaries of what is possible in predictive healthcare.

## Risk quantification

Quantifying the risk of DKD progression in patients with diabetes has been enhanced by advanced algorithms utilizing real-world and electronic health records, demonstrating improved accuracy and clinical applicability over traditional methods.

A pivotal study analyzed the electronic health records data of 417,912 diabetic individuals from the IBM Explorys database and compared the effectiveness of algorithms derived from real-world data with those based on clinical data for quantifying the risk of CKD as a long-term complication of diabetes [7]. The study demonstrated that predictive analytics derived from real-world data can match or even exceed the accuracy of models based on clinical trial data. The results from this study highlighted the robustness of a data-driven and medically informed feature selection strategy. In a related effort, researchers concentrated on the quantification of risk for DKD progression by developing and validating a machine learning risk score that integrated biomarker data with electronic health records information [8]. Utilizing plasma samples from biobanks at Icahn School of Medicine at Mount Sinai and the Penn Medicine, and electronic health records data from individuals with T2D, the research team employed a random forest algorithm to create the KidneyIntelX risk score. This score outperformed traditional models, including the KDIGO risk categories, by showing significant improvements in discrimination metrics such as the AUC and net reclassification index (NRI). Additionally, the risk score increased the positive predictive value (PPV) compared to the KDIGO risk strata. Aligning with national health initiatives like the Advancing American Kidney Health by the US Department of Health and Human Services, this research underscores the importance of accurate risk quantification in preventing or slowing DKD progression and promoting patient-centered kidney replacement therapies.

These studies collectively demonstrate the transformative potential of integrating advanced machine learning algorithms with extensive real-world and electronic health records data for risk quantification in DKD. They signify a substantial shift towards more personalized and precise risk assessment, providing new insights and promising strategies for improving patient outcomes.

## Early detection

Artificial intelligence can aid in the early detection of DKD complications and comorbidities, facilitating timely screening protocols and interventions to mitigate disease progression.

A notable example is the use of ECG-based deep learning models, which have demonstrated considerable promise in identifying renal impairment [9, 10]. Holmstrom et al. [11] conducted an extensive evaluation of their 12-lead ECG-based model, which accurately identified all stages of CKD, particularly in patients under 60 years old. Their research also delved into the model's efficacy in high-risk groups, such as those with diabetes, hypertension, and the elderly, revealing favorable discrimination accuracy in detecting DKD. The model's performance in patients with albuminuria, supported by corresponding laboratory tests and documented estimated glomerular filtration rate (eGFR), highlights its potential as a non-invasive and accessible tool for early DKD detection. In another recent study from China, a new machine learning algorithm was developed to enhance the diagnosis of DKD in patients with T2D, leveraging both retinal vascular parameters extracted from fundus images and easily accessible clinical data [12]. The algorithm utilized a random forest classifier, chosen for its optimal performance compared to alternative classifiers, following an assessment of various methodologies. In a comprehensive validation process, the algorithm exhibited remarkable accuracy, sensitivity, specificity, F1 score, and area of 84.5%, 84.5%, 84.5%, 0.845, and 0.914, respectively.

The ECG-based deep learning model and the integration of retinal vascular parameters with clinical data both exemplify how non-invasive, data-driven approaches can significantly enhance early detection of DKD and help in screening of patients at risk.

## Biomarker identification

Identifying biomarkers for DKD has advanced through the use of machine learning and metabolomics, uncovering novel indicators missed by traditional methods and improving prediction accuracy.

A notable study from the University of Tokyo employed non-targeted metabolomics on plasma and urine samples from DKD patients with an eGFR between 30 to 60 mL/min/1.73 m^2^ [13]. Based on over 30 months of tracking changes to identify rapid kidney function decline, traditional analysis identified urinary 1-methylpyridin-1-ium (NMP) as a promising biomarker. However, the application of deep learning techniques revealed a broader spectrum of potential biomarkers and physiological parameters. Starting with 3,388 variables, researchers narrowed these down to 50 key markers using two regression models to assess their combined utility. The deep learning approach uncovered several critical biomarkers, including systolic blood pressure, urinary albumin-to-creatinine ratio, identified metabolites, and unidentified metabolites like urinary 1-methylpyridin-1-ium. Another study exploring the importance of combining accuracy and stability in feature selection for DKD used an ensemble feature selection framework on a cohort from the University of Kansas Medical Center’s integrated clinical data repository [14]. The authors found that many of the features identified by their framework were not previously considered in the literature. For example, they found that serum anion gap, drug-drug interactions, and Surescript encounters were all associated with DKD risk. They also found that some counterintuitive relationships, such as the inverse relationship between diastolic blood pressure and DKD risk, were also captured by their model. A recent study used machine learning to identify serum myoglobin as a promising biomarker for DKD [15]. By analyzing electronic health records data of 728 hospitalized T2D patients, the researchers developed accurate prediction models that highlighted elevated serum myoglobin levels as a significant predictor of increased DKD risk and renal impairment due to metabolic syndrome components.

## Novel drug target identification

Translating molecular insights from systems biology and omics technologies into effective treatments has been a significant challenge, primarily due to the complex interactions of biomolecules in disease. Researchers have observed that the success of FDA-approved drugs might be attributed to specific properties of their target proteins. By leveraging these properties, machine learning can help predict new drug targets.

A study utilizing a diabetic nephropathy mouse model identified microRNA signatures and potential drug targets using a combination of microarray profiling and qPCR confirmation [16]. The researchers discovered 13 differentially expressed microRNAs in the kidney cortex and six in the medulla. By constructing microRNA-target interaction networks and conducting enrichment analysis, key signaling pathways were identified. To develop a strategy for drug target prediction, the human proteome was annotated with biochemical characteristics and network topology parameters, including proteins targeted by FDA-approved drugs. A novel machine learning algorithm, mGMDH-AFS, was developed to predict drug targets, achieving high accuracy and identifying candidates like Egfr, Prkce, clic5, Kit, and Agtr1a. This approach combines experimental and computational methods, offering new therapeutic targets for DKD.

## Current and emerging commercially available AI solutions for diabetic kidney disease

The field of kidney medicine has experienced significant advancements through the integration of AI which has direct or indirect implications for DKD. Various entities dedicated to incorporating AI into the care of CKD including DKD, have emerged or are currently being developed. Renalytix AI, for instance, has created KidneyIntelX, an AI-enabled clinical decision aid that combines predictive blood-based biomarkers (such as TNF receptor-1, TNF receptor-2, and kidney injury molecule-1) with electronic health records data based on the study by Chan et al.[8]. This innovative model has demonstrated a predictive accuracy surpassing existing clinical models in forecasting DKD progression. Its recent approval and Breakthrough Device designation from the FDA underscores the potential of KidneyIntelX in diagnosing and managing rapidly progressing kidney disease in individuals with Type II diabetes.

Furthermore, pioneering work by organizations like pulseData has led to patents for machine learning systems applied to kidney disease management (Cha et al., 2019, US patent number US20200005900A1). These systems employ AI techniques to generate risk scores using a wide range of data, including demographics, vitals, diagnoses, procedures, diagnostic tests, biomarkers, genetic tests, and patient behaviors or symptoms. Key biomarkers such as eGFR, urine albumin-to-creatinine ratio, and serum creatinine are evaluated, enabling accurate predictions for kidney failure and incident CKD. These models exhibit impressive discrimination, with C statistics exceeding 0.90 for 1-year predictions, further highlighting their potential in DKD risk assessment.

Artificial intelligence has also made substantial contributions to monitoring kidney function and identifying individuals at risk of CKD, including DKD. Google Health’s Kidney Health app, leveraging AI, plays a significant role in these areas. Collaborative efforts between Alphabet's DeepMind and the Department of Veterans Affairs have resulted in considerable progress in predicting acute kidney injury, and probably DKD as well, using AI. By utilizing machine learning software trained on a large cohort of Veterans Affairs patients' medical records, the system demonstrates the capability to forecast the condition up to 48 h in advance, potentially enabling timely interventions to prevent kidney damage[17]. Although the specific focus is not on DKD, this tool holds promise for patients with diabetes who may experience acute kidney injury.

In addition to predictive models, other AI-based technologies hold potential for patients with DKD. Tech giants like Google, IBM, and OpenAI have created medical Chatbots that offer patients access to information about DKD and effectively address their queries. Virtual reality (VR) programs provide an immersive educational experience, equipping patients with a better understanding of DKD and empowering them in self-management.

## Challenges of AI research in diabetic kidney disease

Although the studies show promising potential in the management of DKD, they also have limitations. Variability in information from electronic medical records and non-uniform intervals between laboratory tests pose challenges. Many studies are conducted in single centers, limiting generalizability. The lack of a clear relationship between DKD progression and medication within short time frames suggests the need for prospective studies. Retrospective designs may not guarantee the same performance in clinical settings, and biases can arise from the use of International Classification of Diseases (ICD) codes. The reaction of clinicians to machine learning models is uncertain. Factors influencing trust include user education, past experiences, biases, and perception towards automation, as well as properties of the AI system such as controllability, transparency, complexity of the model, and associated risks. Reliability is a particular concern in healthcare, as changes in AI reliability with new data and the potential for biased or overfitted outcomes can hinder user trust and acceptance of AI systems [18]. Another limitation is the potential for publication bias, where studies with positive outcomes are more likely to be published, possibly skewing the overall perception of AI's performance. This bias can result in an overly optimistic view of AI's capabilities and overlooks studies that may show less favorable or negative results. Further research should involve prospective clinical practice and evaluation of machine learning algorithms to validate their effectiveness in DKD management.

## Transformative potential and future directions of AI-driven research in diabetic kidney disease

Artificial intelligence-driven research for DKD offers a promising future that extends beyond the development of prediction models. Artificial intelligence in Diagnostic and Imaging Technologies enhances the accuracy and efficiency of diagnostic tools. As mentioned earlier, machine learning models based on non-invasive methods like retinal vascular parameters in fundus images combined with clinical data can predict DKD with high accuracy [12]. Kitamura et al. [19] demonstrated that deep learning can aid in the early diagnosis of DKD by analyzing immunofluorescent images of renal biopsies, identifying subtle features not easily detectable by human eyes in light microscopy. This capability allows AI to differentiate DKD from other renal conditions, even when traditional microscopy falls short. Additionally, another study showed that comprehensive nursing intervention, guided by AI-integrated ultrasound technologies, enables dynamic monitoring of renal blood flow and structural changes in real-time [20]. This approach significantly improved renal function and quality of life in DKD patients.

Another crucial area where AI can make a significant impact is the integration of genetic and epigenetic data for the clinical interpretation of DKD, which advances our understanding of the molecular basis of the disease, personalizes treatment strategies, and fosters the development of novel drugs. Machine learning is increasingly uncovering patterns within vast genetic and epigenetic datasets that contribute to the development of diseases. Research indicates that genes associated with DKD are regulated not only by traditional signaling pathways but also by epigenetic mechanisms like chromatin histone modifications, DNA methylation, and non-coding RNAs [21]. For instance, a recent study published in Nature Communications has revealed a correlation between DNA methylation patterns and the decline of renal function in DKD patients [22]. Machine learning techniques can aid in analyzing and interpreting large-scale epigenetic data, facilitating epigenome-wide association studies and the integration of diverse omics data to unravel complex regulatory networks and deepen our understanding of the underlying epigenetic mechanisms in biological processes and diseases. However, further research is needed to demystify the inner workings of deep learning methods, ensuring clinicians can confidently utilize these tools to make informed decisions.

Current pharmacological treatments, including sodium–glucose cotransporter-2(SGLT2) inhibitors, renin–angiotensin system inhibitors, glucagon-like peptide-1 (GLP-1) agonists, nonsteroidal mineralocorticoid receptor antagonists, and combination therapies have demonstrated potential in slowing CKD progression and improving cardiovascular outcomes, but their effectiveness varies among individual patients [23]. By leveraging machine learning and delving into vast patient datasets, future research can identify unique correlations and associations between patient characteristics and treatment responses. Personalized modeling, a promising approach to precise risk estimation, constructs an estimation model for each patient based on a cohort of similar patients, optimizing the model for the individual patient's unique characteristics rather than relying on average patient data [24]. By incorporating personalized modeling into DKD management, healthcare professionals can enhance the precision, accuracy, and equity of risk estimation, ultimately leading to improved patient outcomes and a more effective approach to personalized medicine.

Another future potential of AI in the management of DKD, particularly in light of the observed renal benefits of newer drugs like SGLT 2 inhibitors and GLP-1 receptor agonists [25], lies in leveraging multiple omics platforms and deep learning models to uncover patient-level disease characteristics and individualized treatment responses. By integrating and analyzing heterogeneous multi-modal data using frameworks like multi-omics variational autoencoders (MOVE), significant drug-omics associations can be identified with higher sensitivity than traditional statistical tests [26]. Furthermore, this approach enables the quantification of drug-drug similarities, assessment of polypharmacy, and the distribution of drug effects across various omics modalities, ultimately paving the way for personalized treatment strategies in DKD management. Expanding these approaches to larger cohorts and incorporating longitudinal multi-omics data can enhance causal inference and enable an individualized analysis of patients, leading to the identification of molecular associations, treatment outcomes, and potential biomarkers for personalized medicine in DKD.

The groundbreaking achievements of AI in drug design and protein structure prediction hold immense potential for managing DKD. Companies like Exscientia have pioneered automated drug design, with first-ever AI-designed molecules even entering clinical trials. Other startups like DeepMind, a unit of Google's AI division, focus on developing new drugs using AI technology. Additionally, AI systems like AlphaFold accurately predict protein structures, facilitating a deeper understanding of the molecular basis of DKD and identifying key biomarkers and therapeutic targets. Artificial intelligence-driven drug design can expedite the development of targeted therapies for DKD, discovering novel molecules with enhanced efficacy and safety profiles.

Artificial intelligence is transforming the management of DKD by improving glycemic control and personalizing diabetes care. Artificial intelligence algorithms accurately predict blood glucose levels, optimize insulin dosages, and tailor treatment plans based on individual patient data. Joshi et al. [27] demonstrated the effectiveness of AI-developed personalized nutrition plans in enhancing glycemic control and metabolic health. Numerous studies (reviewed in [28]) highlight the benefits of integrating AI into medical devices and digital platforms for real-time, data-driven diabetes management. These studies show that AI significantly enhances patient engagement and self-management by improving adherence to treatment plans and fostering effective self-care.

In clinical decision-making, AI aids healthcare professionals in making more informed, precise, and tailored decisions, significantly improving care quality for diabetic patients. Artificial intelligence provides deeper insights, predictive capabilities, and personalized treatment strategies, elevating the standard and effectiveness of diabetes care. By sifting through patient data and suggesting the most effective treatment options, AI enhances the accuracy and efficiency of clinical decisions, acting as a powerful ally for healthcare professionals.

## Conclusion

**Fig. 2:**
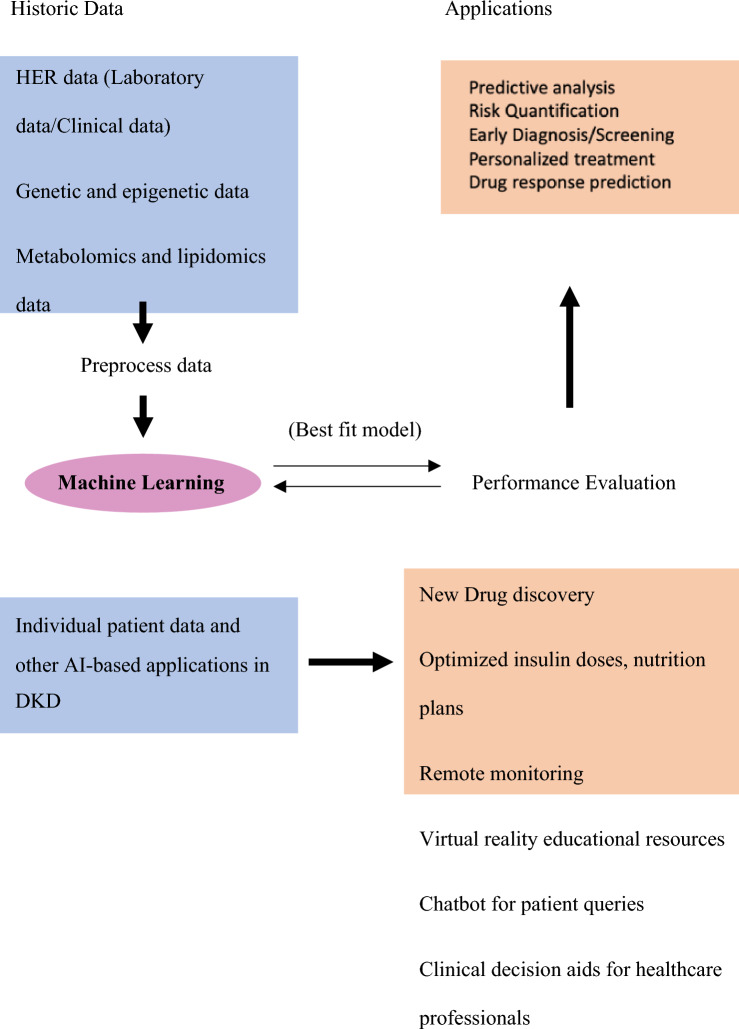
Overview of how machine learning can help manage Diabetic Kidney Disease

In conclusion, future diabetic kidney disease management can greatly benefit from externally validated and broadly applicable AI models that utilize routinely collected laboratory values, clinical data, genomic, transcriptomic, and epigenomic data (Fig. 2). By incorporating risk-based information into electronic health record systems, knowledge can be effectively communicated to healthcare providers and patients, while ensuring continuous calibration, fairness, and external validation in diverse populations. With the aging population and growing healthcare costs, AI algorithms can aid in personalized medicine, improving risk stratification and resource allocation for more efficient and effective care.

## Data Availability

No new data were created or analyzed in this study. Data sharing does not apply to this article.
